# Comprehensive treatment strategy for pancreaticopleural fistula: A rare case report and review of 91 cases

**DOI:** 10.1097/MD.0000000000041184

**Published:** 2025-01-03

**Authors:** Chengsi Zhao, Weijie Yao, Zuozheng Wang

**Affiliations:** a Department of Hepatobiliary Surgery, General Hospital of Ningxia Medical University, Yinchuan 750004, China.

**Keywords:** pancreaticopleural fistula, treatment

## Abstract

**Rationale:**

Pancreaticopleural fistula (PPF) is a rare but serious complication of pancreatic disease, typically resulting from the rupture of a pancreatic pseudocyst or ductal injury. The condition often leads to misdiagnosis due to its nonspecific clinical manifestations, including dyspnea and chest pain.

**Patient concerns:**

A 61-year-old male with a history of alcohol and tobacco use presented with severe dyspnea, chest pain, and cough. He had been diagnosed with acute pancreatitis 9 months prior and intermittently experienced upper abdominal pain and distension post-treatment.

**Diagnoses:**

PPF.

**Interventions:**

The patient underwent thoracic drainage, nasopancreatic duct drainage, and pancreatic duct stent placement, along with parenteral nutrition and somatostatin therapy.

**Outcomes:**

Treatment resulted in resolution of pleural effusion and pseudocyst. The patient had no recurrence during a 5-year follow-up period.

**Lessons:**

This case demonstrates the effectiveness of a comprehensive treatment strategy combining thoracic and pancreatic drainage for PPF. Long-term follow-up is crucial for monitoring recurrence and assessing treatment efficacy. Future research should focus on optimizing treatment plans, particularly regarding the best timing for intervention and improving long-term outcomes.

## 1. Introduction

Pancreaticopleural fistula (PPF) is a rare but serious complication of pancreatic disease. PPF is usually caused by the rupture of a pancreatic pseudocyst or pancreatic duct injury, leading to the leakage of pancreatic enzymes and other secretions into the pleural cavity through the fistula tract.^[1]^ Due to the diverse and nonspecific clinical manifestations of PPF, it often leads to misdiagnosis and delayed treatment.^[2]^ The incidence of PPF in patients with chronic pancreatitis is about 0.4% to 2.49%,^[3,4]^ and it has also been reported in patients with acute pancreatitis.^[5]^ Typical symptoms of PPF include dyspnea, chest pain, cough, and a large amount of pleural effusion, which are often confused with other respiratory diseases, increasing the difficulty of diagnosis.^[6,7]^

Previous studies have explored various treatment methods for PPF, including conservative treatment, endoscopic treatment, and surgical treatment. Conservative treatment typically involves fasting, parenteral nutrition, and the use of somatostatin analogs (such as octreotide) to reduce pancreatic secretion.^[4,8]^ Endoscopic treatment mainly involves endoscopic retrograde cholangiopancreatography (ERCP) for pancreatic duct stent placement to drain pancreatic secretions and promote fistula healing.^[9,10]^ Surgical treatment is often used for cases where conservative and endoscopic treatments fail, with common surgical methods including pancreatic pseudocyst drainage and pancreatic resection.^[11,12]^ These studies not only summarize the efficacy of different treatment methods but also provide practical guidance for the clinical management of PPF. The advantages and disadvantages of different treatment methods and their indications are also focal points of research. Literature indicates that endoscopic treatment has a high success rate in treating PPF, especially for complex cases that cannot be controlled by conservative treatment.^[13,14]^ Although existing studies provide valuable insights, more research is needed on long-term prognosis and determining the optimal timing for treatment.

This article aims to explore the diagnostic process and treatment strategy of a rare PPF case by describing it in detail and analyzing the effects and prognosis of different treatment methods based on our statistical data from PPF-related literature over the past decade. The reported case is a 61-year-old male patient with a complex disease course, treated with a comprehensive strategy combining pancreatic drainage and thoracic drainage, with no recurrence observed over a 5-year follow-up period. The treatment methods and outcomes of this case have certain representativeness and innovativeness in existing literature.

## 2. Case presentation

**Figure 1.**
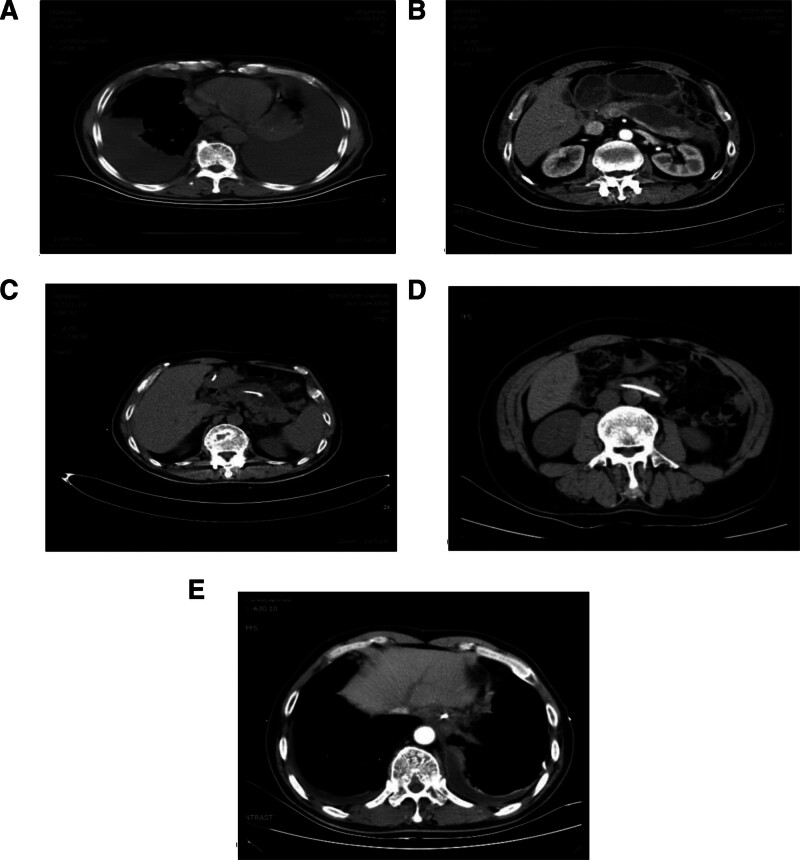
(A–F) Illustrate the patient’s bilateral massive pleural effusion upon admission (A), the formation of a pancreatic pseudocyst (B), guidewire entering the pseudocyst during ERCP (C), disappearance of the pseudocyst 1 week after nasopancreatic duct drainage (D), and resolution of pleural effusion (F). ERCP = endoscopic retrograde cholangiopancreatography.

The patient, a 61-year-old male, was diagnosed with acute pancreatitis 9 months before admission and received conservative treatment. After discharge, he intermittently experienced upper abdominal distension and pain, and an abdominal computed tomography (CT) scan showed the formation of a pancreatic pseudocyst (Fig. 1). Four days before admission, the patient developed severe dyspnea with chest pain and cough, severe enough to prevent lying flat. Physical examination indicated shortness of breath, bilateral diminished breath sounds, tachycardia, and with no abdominal abnormalities. The patient had a long history of alcohol and tobacco use and multiple previous surgeries, including lumbar fixation surgery and abdominal wall lipoma removal.

**Figure 2.**
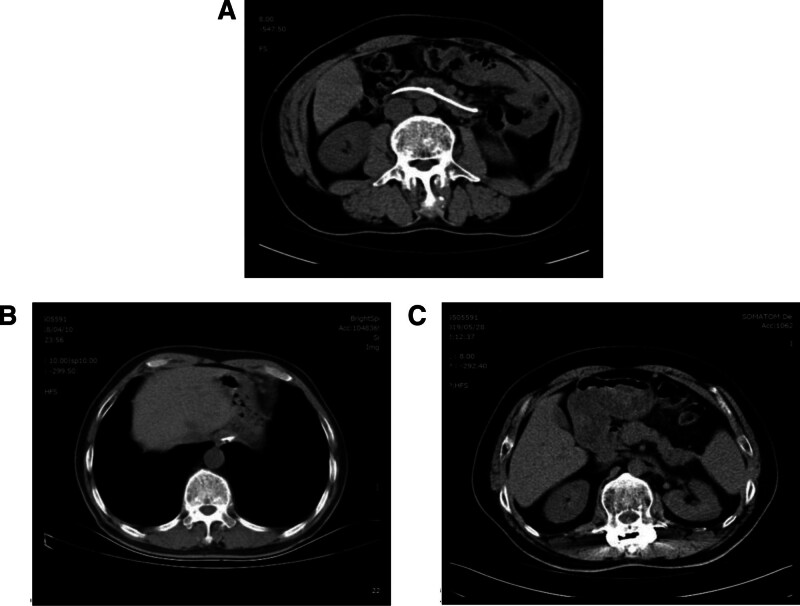
(A–C) Show that 6 months later, the pancreatic morphology of the patient had returned to normal (A), and the pleural effusion had completely disappeared (B), prompting the removal of the remaining nasopancreatic duct. At the 1-year follow-up, the patient had recovered well with no pancreatic complications (C).

Laboratory tests showed slightly elevated serum tumor marker cancer antigen 125 at 98.25 U/mL (normal range 0–35 U/mL) and urine amylase at 333 U/L (normal range 32–641 U/L). Imaging (CT) revealed massive bilateral pleural effusion and the formation of a pancreatic pseudocyst (Fig. 2).

## 3. Diagnosis and interventions

Upon admission, thoracic puncture drainage was performed, bacterial culture of the pleural fluid was negative, no tumor cells were found, but the cancer antigen 125 level in the pleural fluid was significantly elevated at 1859 U/mL (normal range 0–35 U/mL). Amylase levels in the pleural fluid were 53,844 U/L on the left and 1365 U/L on the right. On the sixth day, a diagnosis of PPF was confirmed, and an ERCP was performed with the placement of a 7Fr nasopancreatic duct to drain the pancreatic pseudocyst.

Immediately after admission, thoracic puncture drainage was performed, draining a large amount of pale blood-tinged pleural fluid, along with parenteral nutrition support and somatostatin treatment, gradually assisting the patient in pulmonary function exercises. On the seventh day, an ERCP examination revealed communication between the pancreatic pseudocyst and the pleural cavity. During pancreatic duct cannulation, the guidewire could enter the pseudocyst, so a 7Fr nasopancreatic duct was placed for drainage. The culture of pancreatic fluid from the nasopancreatic duct grew *Enterococcus faecium* and *Klebsiella pneumoniae*, but the patient did not develop fever, and white blood cell count and C-reactive protein were normal, yet antibiotics were administered. On the ninth day, no fluid was drained from the bilateral thoracic drains, and another ERCP was performed. The nasopancreatic duct was cut at the duodenal papilla, leaving the internal part as a pancreatic duct stent.

## 4. Outcomes

The patient received a total of 13 days of hospital treatment, during which 4180 mL of pleural fluid was drained from the left side, 2470 mL from the right, and 495 mL from the nasopancreatic duct. Six months after discharge, the remaining nasopancreatic duct was removed. There was no recurrence during a 5-year follow-up, and the patient’s general condition was good (Fig. 2).

## 5. Literature review

This study statistically analyzed articles on PPF from the past decade, sourcing data from databases such as PubMed and Web of Science, including 58 relevant articles. Inclusion criteria were cases with a confirmed diagnosis of PPF, detailed clinical and treatment data, published in science citation-indexed journals. Data extracted from each article included the number of cases, age, gender, etiology, symptoms, side and volume of pleural effusion, site of pancreatic duct rupture, serum amylase level, treatment methods and outcomes, and follow-up duration (Table 1).

**Table 1:** Clinical characteristics and treatment status of PPF in relevant literature in the past decade.

Item	Content
Number of cases	91 cases
Regional distribution	Asia: 34 (37.36%), Europe: 37 (40.66%), Americas: 18 (19.78%), Oceania: 2 (2.20%)
Gender ratio	Male: 77 (84.62%), female: 14 (15.38%)
Age range	3–81 years
Median age	51 (41–58) years
Main symptoms	Dyspnea: 68 (74.73%), chest pain: 42 (46.15%), cough: 11 (12.09%), abdominal pain: 29 (31.87%), fever: 14 (15.38%)
Symptom characteristics	Single symptom: 32 (35.16%), multiple symptoms: 59 (64.84%)
Major etiologies	Chronic pancreatitis: 71 (78.02%), acute pancreatitis: 7 (7.69%), tumor: 2 (2.20%), traumatic pancreatitis: 2 (2.20%), genetic mutation: 3 (3.30%)
Combined etiologies	Pseudocyst: 27 (29.67%), alcoholic pancreatitis: 48 (52.75%)
Side of pleural effusion	Left: 52 (57.14%), right: 31 (34.07%), bilateral: 8 (8.79%)
Volume of pleural effusion	Large: 85 (93.41%), moderate: 6 (6.59%)
Site of pancreatic duct rupture	Tail: 28 (30.77%), body: 36 (39.56%), head: 20 (21.98%), body and tail: 3 (3.30%), neck: 2 (2.20%)
Treatment methods	Endoscopic: 71 (78.02%), conservative: 3 (3.30%), surgical: 17 (18.68%)
Treatment duration	21 d (IQR: 14–31.75 d)
Pleural drainage duration	12 d (IQR: 7–14 d)
Pancreatic duct stent duration	42.0 d (IQR: 14.0–90.0 d)
Follow-up duration	6.0 mo (IQR: 3.0–12.0 mo)
Recurrence	2 (2.20%)

IQR = interquartile range, PPF = pancreaticopleural fistula.

In total, 58 articles covering 91 PPF cases were included. Here is an overview of the specific data (Table 1).

## 6. Discussion

PPF is a rare but serious complication of pancreatic disease, usually caused by the rupture of a pancreatic pseudocyst or pancreatic duct injury. This paper describes a case of a 61-year-old male patient with PPF. PPF is more common in males, accounting for 84.62% of cases in the literature we reviewed, possibly related to the more prevalent history of smoking and drinking in males, which are known risk factors for chronic pancreatitis.^[15]^ Chronic pancreatitis is the primary cause of PPF, confirmed in 78.02% of cases in our literature review. Previous literature reported that about 67% of PPF cases occur in alcohol-related chronic pancreatitis.^[16]^ In the past decade, 52.75% of PPF cases occurred in alcoholic pancreatitis. A small number of PPF cases are caused by traumatic pancreatitis, autoimmune pancreatitis, and genetic mutations. The main reason for PPF formation is the rupture of a pseudocyst in the pleural space.^[17]^ Therefore, PPF often coexists with pancreatic pseudocysts, as seen in our case. Approximately 29.67% of PPF patients in the past decade had pancreatic pseudocysts, primarily caused by pancreatic duct rupture in the body of the pancreas (39.56%), followed by the tail (30.77%). This suggests that when placing a pancreatic duct stent to treat PPF, it is best to place the stent at the tail of the pancreas to reduce pancreatic fluid leakage. Because pancreatic duct rupture in the body and tail of the pancreas is more common, most PPF patients have left-sided pleural effusion. Previous case series and literature reviews show that about half of the diagnosed patients present with left-sided pleural effusion, with a bilateral effusion rate of 14% to 16%.^[18,19]^ In the past decade, 57.14% of PPF cases in the literature had left-sided pleural effusion, with only 8.79% having bilateral effusion. Our case was rare, with bilateral massive pleural effusion. The primary reason for left-sided pleural effusion is the anatomical position of the pancreas near the left pleura, esophagus, and aortic hiatus. In acute pancreatitis, pancreatic fluid tends to drain into the retroperitoneal and extraperitoneal spaces of the pelvis.^[20]^ Such pleural effusion is often massive, making chest symptoms the first to appear in PPF patients. Our literature review found that dyspnea is the most common symptom (74.73%), with about half of the patients experiencing chest pain. Other symptoms include cough, abdominal pain, and fever, with 40.59% of patients having multiple chest symptoms. Such clinical manifestations often lead to delayed diagnosis of PPF, and PPF should be considered in patients with pleural effusion and a history of pancreatitis.

There is still no established optimal treatment plan for PPF, but basic conservative treatment is essential. Studies suggest that fasting and inhibition of pancreatic secretion can reduce pleural effusion production at the source.^[21–23]^ However, 59% to 69% of patients with simple conservative treatment cannot achieve fistula closure. Prolonged fasting can lead to intestinal mucosal atrophy, sepsis, and malnutrition, so promoting fistula closure as soon as possible is critical.^[16,24]^

In 1993, Saeed et al^[25]^ first reported endoscopic pancreatic stent placement for treating pancreaticopleural fistula. The stent’s role is to drain and bridge the rupture site of the pancreatic duct, promoting fistula closure.^[26–28]^ Pancreatic stents are used in treating complications of chronic pancreatitis and pseudocysts in acute pancreatitis, which are the main causes of PPF.^[29,30]^ Pancreatic stent placement can also relieve abdominal pain in patients with chronic pancreatitis. Additionally, pancreatic stents help prevent post-ERCP pancreatitis, so PPF patients undergoing ERCP should routinely have a pancreatic stent placed, whether or not bridging can be achieved, to reduce pancreatic duct pressure and prevent complications.

In our case, nasopancreatic duct drainage was used. Compared to pancreatic stents, nasopancreatic ducts have the advantage of preventing obstruction through suction. The drainage effect and volume can be directly observed to determine if the fistula has closed. Repeated pancreatic imaging can confirm fistula closure without needing repeated ERCP.^[31]^ For patients with infections, nasopancreatic duct drainage can facilitate bacterial culture of pancreatic fluid to determine if the pancreas is infected and identify pathogens, preventing severe complications. Nasopancreatic ducts have advantages in observing drainage effects and preventing obstruction, suitable for complex cases. Nasopancreatic ducts cause more discomfort than pancreatic stents. In our case, once the pleural effusion no longer increased and there were no infectious complications, the nasopancreatic duct was cut at the duodenal papilla, converting it into a pancreatic stent. Alternatively, the nasopancreatic duct can be replaced with a pancreatic stent after 1 week. Some studies suggest replacing the nasopancreatic duct with a pancreatic stent after 1 week,^[32,33]^ but we believe this should be decided based on the patient’s specific condition. Electrolyte balance and fluid supplementation should be monitored in patients with nasopancreatic duct placement.

Existing reports show that the cure rate of endoscopic treatment for PPF ranges from 50% to 86.36%. Previous studies suggested that endoscopic and other minimally invasive treatments should be used after conservative treatment fails.^[34]^ We performed ERCP drainage immediately after diagnosing PPF, believing this does not conflict with conservative treatment and effectively reduces pancreatic fluid entering the pleural cavity, alleviating symptoms and promoting healing. Recent literature also shows that 78.02% of patients used endoscopic treatment. Clearly, endoscopic treatment has become the primary method for PPF in recent years. The main advantage of endoscopic treatment is placing stents to cover pancreatic duct rupture sites and dilate strictures through minimally invasive methods, achieving decompression and healing.^[8,35]^ Endoscopic stent placement can usually close the fistula within 2 to 3 weeks, with stents remaining in place for 4 to 6 weeks, significantly shortening hospital stays.^[36,37]^ Overall, endoscopic treatment is suitable for complex cases or those that failed conservative treatment, especially PPF caused by chronic pancreatitis. It is unsuitable for patients with severe pancreatic duct obstruction or rupture sites inaccessible by endoscopic methods. Early endoscopic drainage is recommended upon PPF diagnosis.

In the past decade, 18.68% of patients still underwent surgical treatment, including those who failed endoscopic treatment. Surgery is undoubtedly the first choice for treatment after endoscopic treatment fails.^[38–41]^ Previous studies suggested that pancreatic duct obstruction and rupture in the pancreatic tail should be treated surgically.^[42,43]^ Although the incidence of pancreatic tail duct rupture in PPF remains high in recent years, most patients still underwent endoscopic treatment. The advantage of surgical treatment is a high success rate of 94%. Surgery can thoroughly solve the problem, prevent recurrence, and have a low complication rate.^[37,44,45]^ The main drawbacks of surgical treatment are significant trauma and numerous complications.^[46,47]^ Overall, surgical treatment is suitable for patients who failed conservative and endoscopic treatments or have severe complications such as complete pancreatic duct obstruction. It is not suitable for patients with high surgical risk or those unable to tolerate surgery.

We conducted a 5-year follow-up for the patient. Recent literature shows an average follow-up duration of 6 months. Follow-up is crucial in managing PPF, primarily for monitoring recurrence, assessing treatment efficacy, preventing and managing complications, providing nutritional support, and psychological support. Regular clinical and imaging examinations (such as CT, magnetic resonance cholangiopancreatography) during follow-up can detect recurrence early and take corresponding measures, improving long-term prognosis. Assessing treatment efficacy and understanding whether the fistula has fully closed helps optimize treatment plans. In preventing complications, follow-up can detect and manage pancreatitis, pseudocysts, and pleural infections early. Nutritional support and lifestyle guidance through follow-up ensure adequate nutrition and support for patients. Psychological support helps patients cope with the psychological challenges brought by the disease. Follow-up can also enhance doctor-patient communication and improve patient compliance with treatment. Specific recommendations are monthly follow-ups after initial treatment, followed by every 3 to 6 months for stable cases, including physical examinations, laboratory tests, and imaging studies.

## 7. Conclusion

By statistically analyzing 106 PPF cases over the past decade, we provide more detailed data on the clinical characteristics and current diagnosis and treatment of PPF. We successfully treated this patient using a comprehensive strategy of thoracic drainage, pancreatic internal and external drainage, and long-term follow-up. The successful treatment of this case provides practical evidence for the comprehensive management of PPF. More research is needed in the future to optimize treatment plans, particularly regarding long-term prognosis and determining the best timing for treatment.

## Author contributions

**Conceptualization:** Zuozheng Wang.

**Data curation:** Chengsi Zhao, Zuozheng Wang.

**Formal analysis:** Chengsi Zhao, Weijie Yao, Zuozheng Wang.

**Investigation:** Chengsi Zhao.

**Methodology:** Chengsi Zhao.

**Writing – original draft:** Chengsi Zhao, Weijie Yao.

**Writing – review & editing:** Weijie Yao.
